# Management of environmental health issues for the 2004 Athens Olympic Games: is enhanced integrated environmental health surveillance needed in every day routine operation?

**DOI:** 10.1186/1471-2458-6-306

**Published:** 2006-12-18

**Authors:** Christos Hadjichristodoulou, Varvara Mouchtouri, Vasiliki Vaitsi, Christina Kapoula, Anastasia Vousoureli, Isidiros Kalivitis, Julia Chervoni, Panagiotis Papastergiou, Antonios Vasilogiannakopoulos, Vasilis D Daniilidis, Jenny Kremastinou

**Affiliations:** 1 University of Thessaly, Faculty of Medicine, Department of Hygiene and Epidemiology, Larissa, Greece; 2 National School of Public Health, Olympic Planning Unit, Athens, Greece; 3 US Centers for Disease Control and Prevention's (CDC) Vessel Sanitation Program (VSP), Atlanta, USA

## Abstract

**Background:**

Management of environmental health issues is an integral part of public health systems. An active integrated environmental health surveillance and response system was developed for the Athens Olympics to monitor and prevent exposure to environmental hazards. The potential for permanent implementation of the program was examined.

**Methods:**

The environmental health surveillance and response system included standardization, computerization and electronic transmission of data concerning environmental inspections of 17 site categories (restaurants, swimming pools etc) of public health interest, drinking and recreational water examinations and suggested corrective actions. The Olympic Planning Unit integrated and centrally managed data from 13 public health agencies, recommended, supervised and coordinated prompt corrective actions. Methods used to test the effectiveness of the program were the assessment of water quality test and inspection results trends over time using linear regression and epidemiological surveillance findings.

**Results:**

Between January 2003 and September the 30th, 2004, 196 inspectors conducted 8562 inspections, collected 5024 water samples and recommended 17 027 corrective actions. In 10 cruise ships used as floating hotels inspectors conducted 10 full inspections, 2 re-inspections, and 27 follow-up inspections. Unsatisfactory inspection results (r = 0.44, p < 0.0001) and positive water quality tests (r = 0.39, p < 0.001) presented an overall decrease trend over time. In August, 2003, an outbreak of salmonellosis was linked to a hotel restaurant which accommodated athletes during a test event.

**Conclusion:**

Lessons learned for future events include timely implementation and installation of communication processes, and rapid and coordinated response to unsatisfactory inspection results. Routine national programs need to adopt enhanced environmental health surveillance aimed at public health decision-making, but with a different perspective.

## Background

A public health sector, enforcing and ensuring prevention, control, and management of environmental health issues, is essential during a mass gathering event such as the Olympic and Paraolympic Games. Approximately 500 000 visitors, 15 000 athletes from 202 countries, and 6900 official media representatives gathered in Athens for the 2004 Olympic Games [1] which started on August 13 and finished on August 29. The Paraolympic Games were scheduled two weeks later, starting on September 17 until September 28. Olympic period was defined as the period between August 2 (opening day of the Olympic Athlete's Village) and September 30 (closing day of the Olympic Venues).

A large number of people were potentially exposed to possible environmental health hazards given the massive food service operations[2,3], common and excessively used potable and recreational water supplies, crowded accommodation, and shared sanitary facilities in hotels, Olympic Venues, and cruise ships. All of these provided conditions favourable to the spread of infectious diseases. Similarly, we can safely say that modern urban lifestyle, featuring massive food production and consumption, crowded accommodation settlements, common water supplies and heavily overloaded sanitation and sewage systems, may resemble the situation of a mass gathering event, thus presenting a substantial smoldering risk for disease transmission. Environmental health issues as part of public health surveillance were effectively managed in previous Olympic Games, [4-9] and other mass gathering [10] while enhancements initiated for the Games were utilized to yield long term benefits for the local public health systems[11].

In 2001, a capacity assessment study documented deficiencies in personnel, resources, equipment, training, and coordination of the Hellenic Public Health Agencies[1]. Doubts about the success of the 2004 Olympic Games had been expressed in the international press, while there were worrying signs that preparations in Athens might fail to deliver in time. Therefore, careful planning and intensive preparations were required to successfully respond to the major challenge of the Olympic Games.

Foodborne and waterborne illnesses, travelers' diarrhea, and airborne diseases were considered the potentially highest public health threats[1]. Based on the capacity assessment findings and data obtained from previous Olympic Games, environmental health issues that were identified as essential in the protection of public health were summarised under the headings of: food safety, water quality (potable, recreational), sanitation, Legionnaires' disease prevention, vessel sanitation, waste management, and vector control[1,12].

In this paper we report how the environmental health surveillance program for the Athens Olympic Games was implemented, we present its results, and discuss the lessons learned. We explore the potential for using enhanced environmental health surveillance not only during mass gatherings, but also within the national routine public health system.

## Methods

An integrated environmental health surveillance program was decided to be implemented including a computerized standardized inspection system of all environmental health aspects and water microbiological quality monitoring. The High-level Olympic Public Health Committee of the Hellelic Ministry of Health approved the program. The main objectives were the early detection of emergencies that might require rapid intervention and the coordination of corrective actions. Environmental health targets included hotels, restaurants, other food premises, canteens, water supply systems of buildings, swimming pools, decorative fountains, cooling towers, passenger ships, camps, seacoasts, airports, marinas, bottled-water plants, ice-producing plants, waste management facilities, sewage treatment units, public toilets, areas requiring pest control, and the Olympic cruise ships. Out of the total 44,741 premises of environmental health interest were identified as potential inspection sites, 5,724 premises were registered and inspected. The methodology and criteria used to choose the premises inspected, and the coverage rate per inspection topic have been published and discussed elsewhere [12]. The personnel of 13 Prefecture Departments of Public Health (PDPH) performed the inspections in the five Olympic cities of Greece (Athens, Thessaloniki, Volos, Iraklio, and Patra).

PDPH did not have jurisdiction on food safety issues within Olympic Venues, therefore food premises within the Olympic Venues were excluded from the environmental health surveillance program. Food premises inside the Olympic Venues were inspected by the personnel of the National Agency for Food Safety. The environmental health surveillance program included food safety issues related with food premises located outside the Olympic Venues.

### Standardized inspections

Registrations and inspections were standardized to ensure uniformity and consistency of procedures as well as efficient electronic management of data. A total of 19 forms were developed to register information regarding premises of environmental health interest (see additional file 1)[12]. Data requested by each registry form included the name and the unique registration code of the establishment, the address including county of jurisdiction, as well as standardized questions to identify typical and representative characteristics of each premise according to the type of the premise (e.g., the swimming pool registry form included questions regarding the capacity of the pool, the method of disinfection, the type of filtration).

A total of 17 standardized scored inspection forms were developed (Table 1) including establishment name, unique code, inspection identification code, county, date of inspection, overall score, specific violations cited, manually tested measurements during inspection (free chlorine, pH, temperatures), inspectors, and time spent on inspection. The results of the inspections were graded as: "A", satisfactory, "B", relatively satisfactory, or "C", unsatisfactory, respectively[12-14].

### Water quality surveillance program

The main objective of the water quality surveillance program was to assess the quality of the water at the point of use. The following seven standardized sample collection forms were developed to standardize sampling procedures: sample collection forms for swimming pools, water distribution systems, bottled-water plants, seacoasts, water distribution systems (*Legionella *spp. detection), cooling towers (*Legionella *spp. detection), and decorative fountains (*Legionella *spp. detection).

Samples collected from water supply systems of Olympic Venues, hotels, and floating hotels were tested for the presence of coliform bacteria, *Escherichia coli*, intestinal enterococci, and *Clostiridium perfringens *(including spores). These microbiological parameters are obligatory for routine monitoring of water quality, according to the Greek and European legislation[15]. In addition, special samplings were conducted in distribution systems to detect and enumerate *Legionella *spp. in water samples.

Swimming pools water samples were tested for the presence of coliform bacteria, *Escherichia coli*, *Staphylococcus aureus*, *Pseudomonas aeruginosa*, and heterotrophic plate count at 37°C[16]. Water samples from swimming pools and water supply systems of Olympic Venues and floating hotels were additionally tested for *Cryptosporidium *spp., *Giardia *spp., and Norovirus.

Water collected from cooling towers and decorative fountains was tested for the presence of *Legionella *spp. and for aerobic counts at 37°C, at a minimum of 48 hours incubation.

The threshold concentrations used to characterize a sample as positive have been published elsewhere[13,14]. The Laboratories performed microbiological testing were participated in an external quality control scheme (EQUASE) before and during the research period. Water samples collected from water supply systems were tested for microbiological parameters in accordance with the methods specified in the standing European legislation[15].

The detection and enumeration of *Legionella *spp. was performed at the *Legionella *Reference Laboratory of the National School of Public Health. Before and during the research period, the reference laboratory participated in an external quality-control scheme for *Legionella *detection in water (Quality Management Ltd, Lancashire, UK), through a periodic distribution of water samples seeded with unknown *Legionella *spp. and concentration.

### Standardized corrective actions

For each deficiency cited during an inspection, a standardized corrective action was suggested in the corrective action form. In addition, standardized guidelines for the disinfection of water distribution systems, cooling towers, swimming pools, and decorative fountains were created by the Olympic Planning Unit and were delivered to the PDPH as a response to every positive laboratory result received. The standardized disinfection guidelines were created according to the European Working Group for *Legionella *Infections Guidelines[17], and the World Health Organization Guidelines[16].

### Computerized electronic network

Using the Epi-Info 2000 software, 19 Geographical Information System databases were developed to record the establishments (registry database), 17 databases to record the standardized inspection forms used including the documented results, and 6 databases to record the microbiological sample test results. A unique code was given to each establishment to relate the registration, inspection, and microbiological results by using this code. Furthermore the Geographical Information System was used to implement an integrated environmental health surveillance program[12].

**Figure 1:**
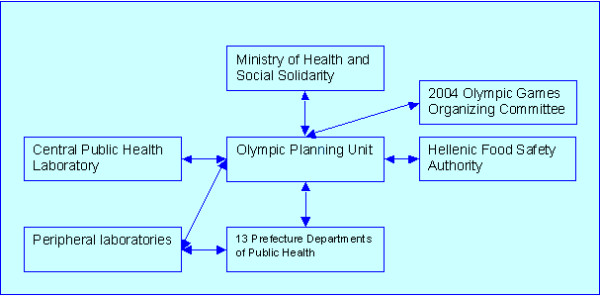
Communication flow chart of the environmental health surveillance and response system for the 2004 Olympic Games.

A virtual private communication network connecting the 13 PDPH, the Central Public Health Laboratory, the Olympic Planning Unit, and the Ministry of Health and Social Solidarity provided the opportunity to collect, collate, analyse, review, and share the results of the environmental health inspections and microbiological tests in real time (Figure 1).

### Training and Guidelines for professionals and owners

Up-to-date training material on food safety, water quality (potable, recreational), water and food sampling, Legionnaires' disease prevention, vessel sanitation, waste management, and vector control was prepared to be used in practical and theoretical training programs of PDPH officers. Special attention was given in standardized inspection training to avoid subjective interpretation during inspections. Training material was also prepared and distributed to facility owners, and the public.

### Vessel Sanitation

Ten cruise ships were contracted by the Athens 2004 Olympic Organizing Committee to be used as floating hotels in the Piraeus Harbor. The Ministry of Health and Social Solidarity identified a lack of a standardized or coordinated response to a potential public health threat for passengers and crew members. Many different authorities were involved with similar, but not clearly assigned responsibilities. Thus, a coordinated approach on ship sanitation and communicable disease surveillance was required.

For the 2000 Sydney Olympic Games, a Vessel Inspection Program was introduced to inspect the ten cruise ships which were moored in the Sydney Harbor and served as floating hotels[18]. The National School of Public Health in collaboration with the Centers for Disease Control Vessel Sanitation Program (CDC VSP), developed the Hellenic Vessel Sanitation Program to ensure the health and safety of passengers and crew members. The Hellenic Vessel Sanitation Program was closely modeled on the CDC VSP[19] which has been successful in reducing diarrhoeal illness on board cruise ships since its introduction in 1975[20]. Based on European Union and Greek legislation and World Health Organization Guidelines, the Hellenic Vessel Sanitation Program defined and enforced requirements regarding potable water safety, swimming pool operation, food safety, sanitation, Legionnaire's disease prevention, waste management, and vector control. Special regulations were created concerning the implementation of the Hellenic Vessel Sanitation Program and applied during the Olympic Games.

### Performance

The overall objective of the program was to prevent outbreaks among athletes and spectators and to have improved scores in standardized inspections and in satisfactory microbiological tests. Statistical analysis over time of the standardized inspection results and water quality tests results together with the communicable diseases surveillance findings were used to assess the performance of the environmental health surveillance program.

Time series analysis was performed to detect association between time and inspection or microbiological test results. The inspection and microbiological test results were categorized in four periods: January through December 2003, January through May 2004, June through July 2004, and the Olympic period (from August through September 2004). The linear regression and chi-square test for trend were also used. All statistical analyses were conducted by using the software package Epi-Info 2000 (CDC, Atlanta, Georgia) and SPSS for Windows Release 11.01.

## Results

### Standardized inspections

A total of 44,741 premises of environmental health interest were identified as potential inspection sites. From January 2003 through July 2004, 196 environmental health inspectors registered via on-site visits a total of 5,724 premises using standardized registration forms.

During the preparation years of 2002, 2003 and 2004, a series of 46 "test events" were held to evaluate venues and logistics. 5,956 inspections were carried out from January 2003 through July 2004 (Table 1). 2606 inspections were carried out during the Olympic Games (from August through September 2004), 1739 of which were performed within the Olympic Venues and 867 outside the Olympic Venues (Table 1). Out of 8562 inspections, 5673 (66.2%) were satisfactory, 1922 (22.4%) were relatively satisfactory, and 967 (11.3%) were unsatisfactory. Inspections presented in Table 1 were repetitive, while some of them conducted in new sites.

### Water quality surveillance program

Out of 4242 water samples collected from January 2003 through July 2004, 995 were collected within and 3247 outside the Olympic Venues (Table 2). During the Olympic period, out of a total of 782 water samples, 571 were collected within the Olympic Venues and 211 outside the Olympic Venues (Table 2). Out of 2497 samples tested for *Legionella *spp, 415 (16.6%) were positive. Out of 1909 samples collected from water supply systems and bottled water plants and tested, 227 (11.8%) were positive. Out of 618 samples collected from swimming pools and seacoasts and tested 76 (12.2%) were positive. None of the 105 samples collected from swimming pools and water supply systems, and tested turned positive for *Cryptosporidium *spp., *Giardia *spp., or Norovirus. Microbiological examinations presented in Table 2 were repetitive, while some of them conducted in new sites.

### Standardized corrective actions

14 714 corrective actions were put into effect, from January 2003 through July 2004, and 2313 corrective actions were undertaken during the Olympic period.

### Computerized electronic network

All data from the registrations and the inspection results were entered into the electronic databases in the PDPH and were delivered to the Olympic Planning Unit. Data from the microbiological test results were entered into the electronic databases in the Central Public Health Laboratory as well as the Peripheral Public Health Laboratories and were delivered to the Olympic Planning Unit and the PDPH. It was the responsibility of the Olympic Planning Unit to relate all data and to produce reports once a day, which were sent to the Ministry of Health and Social Solidarity and the Athens 2004 Olympic Games Organizing Committee (Figure 1). The private virtual electronic communication network operated 18 hours daily and allowed rapid distribution of information. No network failure occurred since its installation. A total of 28 daily reports were sent during the Olympic Games and 12 during the Para Olympic Games. The daily reports included the total number of "A", "B", or "C" grading inspection results, the number of positive or negative microbiological test results, and the number of suggestive corrective actions, for each PDPH. An additional highly detailed daily report was created including all unsatisfactory ("C" grading result), or positive microbiological test results. The compliance deadline for the facility owners was no more than 24 hours. The follow-up inspection results were sent to the Olympic Planning Unit through the electronic network, immediately after performing the inspections.

### Training and Guidelines for professionals and owners

From January 2003 through February 2004, 865 hours of training were conducted. A total of 196 environmental health inspectors underwent uniform training and certification in order to carry out consistent standardized inspections and water sampling. Moreover, a series of short training courses concerning epidemiological investigation, vector control, ship sanitation, response to emergencies and disasters, and Epi-Info software were attended by 250 PDPH officers including the 196 environmental health inspectors of the Olympic cities.

A series of guidelines were published and distributed to the environmental health inspectors and facility owners: 5000 copies providing guidelines for safe operation of swimming pools, 5000 copies of guidelines for *Legionella *prevention in cooling towers, water systems, and decorative fountains, 5000 copies of guidelines for water sampling procedures, and 5000 copies of shipboard pest management manuals. Approximately 1 000 000 copies providing instructions for safe swimming in Greek and English were distributed to the public.

### Vessel Sanitation

On arrival, the 10 cruise ships used as floating hotels underwent a full vessel inspection by a group of inspectors consisting of officials and experts from the Ministry of the Mercantile Marine, the Veterinary Directorate of the Piraeus Prefecture, the National School of Public Health and the CDC VSP. After the inspection, the vessels were given an inspection score according to the Hellenic Vessel Sanitation Program. Two of them were presented with "unsatisfactory" grading results and were re-inspected after corrective actions were implemented. A total of 27 follow-up inspections were carried out every three to four days concentrating on food buffet services (in particular food temperatures), water supply, and swimming pool and spa maintenance.

At the same time, microbiological testing was conducted in 45 samples collected from drinking water tanks, ice-making machines, and kitchen water taps, and 30 samples originating from swimming and spa pools. During inspections, spoiled or expired food items were found and destroyed on three cruise ships; water-related problems were associated with four cruise ships. *Pseudomonas aeruginosa *was detected in samples collected from the swimming pools of four vessels.

### Performance

An outbreak of salmonellosis among the German junior rowing team, which prevented them from participating at the respective rowing championship test event, occurred in August, 2003. The restaurant of the hotel accommodating the team, which was linked to the outbreak, had received one of the lowest unsatisfactory inspection results seven days before the outbreak occurred. No other disease outbreak was recorded by the enhanced communicable diseases surveillance system before or during the Olympic Games within the Olympic cities[21,22].

**Figure 2:**
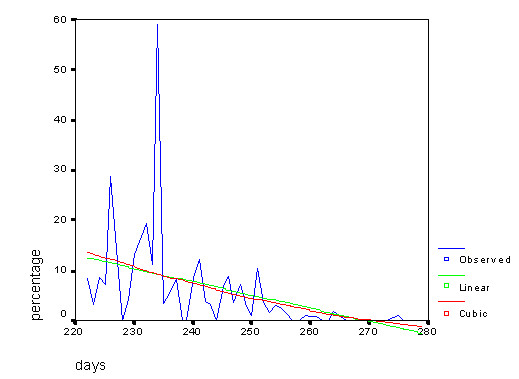
Distribution and regression of the percentage of unsatisfactory inspection results of premises inspected outside the Olympic Venues 160 days before the end of the Olympics, r = 0.44, p < 0.0001.

**Figure 3:**
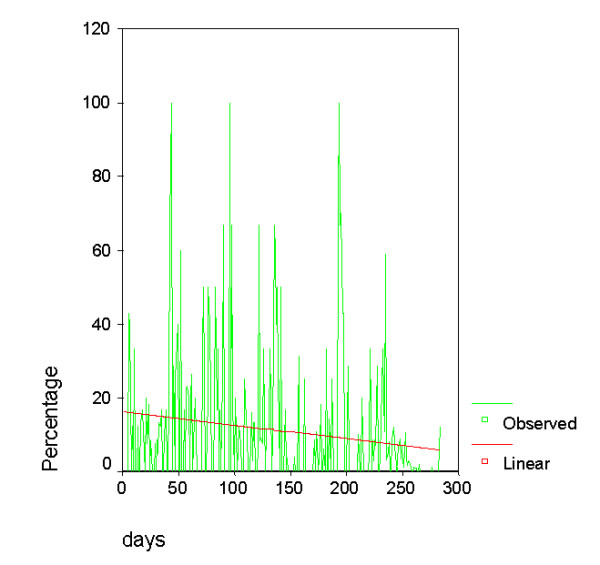
Distribution and regression of the percentage of unsatisfactory inspection results of premises inspected within the Olympic Venues 300 days before the end of the Olympics, r = 0.16, p < 0.001.

**Figure 4:**
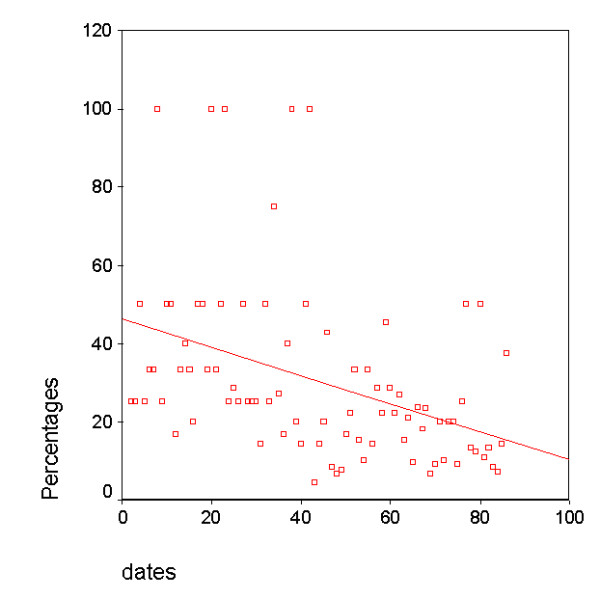
Regression of the percentage of positive microbiological test results 100 days before the end of the Olympics, r = 0.397, p < 0.001.

An overall decrease trend in unsatisfactory inspection results (Figure 2, r = 0.44, p < 0.0001, and Figure 3, r = 0.16, p < 0.005) as well as positive water quality tests (Figure 4, r = 0.39, p < 0.001) was noted during the pre-Olympic and Olympic period. As is shown in Figures 2, 3, and 4 a periodical increase and decrease of unsatisfactory results is noted, which is related to the starting days of the test events where inspectors conducted intensive inspections and sampling. Microbiological results are presented during the last 100 days before the end of the Olympics, because in this period were conducted most of the sampling in everyday basis. The same for the figure 4, in which 160 days before the end of the Olympics were conducted most of the inspections outside the Olympic venues. Figure 3 includes the results of the last 300 days before the end of the Olympics because a large number of inspections were conducted inside the venues during test events, which were taken place over one year period before the Olympics. The percentage of unsatisfactory inspection results during the first period of inspections was 22.8% (339), whereas only 3.7% (96) of the last inspections, before the Olympic Games, presented unsatisfactory results (Table 1).

During the first sampling, positive microbiological test results were confirmed in 117 (12.4%) of the samplings, whereas during the last sampling before the Olympic Games 102 (13%) of the samplings were positive. Positive microbiological test results remained quite stable from January 2003 through May 2004, increased during June and July of 2004 and declined during the Olympic period (Table 2).

The 223 hotels that were contracted by the Athens 2004 Olympic Organizing Committee to be used as accommodation sites received satisfactory inspection results in the last inspection before the Olympic Games. 76 (34%) out of the 223 hotels had received "unsatisfactory" or "relatively satisfactory" inspection grading results during the first inspection, and they had a mean improvement of 9.2 points during the last inspection before the Olympic Games. During the first water sampling of the 223 hotels, 232 (14.6%) out of the 1584 samples collected turned positive. No positive water microbiological test result was reported during the last microbiological test before the Olympic Games.

## Discussion

During the summer of 2004, the entire world shared the power and magic of the Olympic and Para Olympic Games, which were carried out with absolute success[23]. Environmental health planning and response contributed to the success of public health aspects of the 2004 Athens Olympic Games. Methods used to deliver environmental health services were assessed for their efficiency and ability to enhance desired public health outcomes. The epidemiological surveillance findings[21,22] and the gradual improvement of the results of the inspections and the water quality tests indicated the effectiveness of the environmental health surveillance program applied, which was based mostly on the corrective actions were taken.

The improvement of inspection results of cooling towers and water distribution systems could be explained by the alertness of hoteliers and associates of the Athens 2004 Olympic Games Organizing Committee achieved through the guidelines concerning Legionnaires' disease prevention. The increase of positive microbiological test results during the last two months before the Olympics may be related to the newly constructed water supply systems of the Olympic Venues. It should be noted that some Olympic Venues were ready for use and sampling just one or two weeks before the Olympic Games, thus, the first sampling microbiological results were not satisfactory since inadequate flushing might have been carried out. The increase of unsatisfactory inspection results of food premises from January through May 2004 may be attributed to the thorough inspections conducted by the inspectors after the outbreak of salmonellosis in August, 2003. The number of inspected seacoasts was small and therefore it is not safe to draw any conclusion.

The Environmental Health Surveillance System deployed for the Athens Olympic Games, was more comprehensive than systems applied during previous Olympic Games. New features included: a) the integration of standardized inspection results of 17 different site categories together with the laboratory results of microbiological tests, b) the highly detailed electronic development of inspection reports directly linked to suggested corrective actions, and c) the application of the Geographical Information System. The system computerization and the electronic network allowed electronic data exchange and production of reports at any time during the day, while data could be compared with epidemiological surveillance findings. The electronic system was able to automatically detect positive microbiological results or premises that failed inspection and schedule counterchecks. Application of emerging technology proved to be useful in environmental health surveillance.

The importance of centralized control and communication, and rapid response capabilities is illustrated by a successful response to early reports of poor food-handling practices[24]. This was not achieved at the 2003 outbreak, but it was corrected thereafter by the communication network. The hotel restaurant mentioned above that was identified as the possible source of a salmonellosis outbreak in August, 2003, had received an unsatisfactory inspection grade one week before the outbreak occurred. The Athens 2004 Olympic Games Organizing Committee had not been informed of the inspection result, since the communication and response structure had not yet been established. Communication mechanisms of Health Departments should involve the Olympic Games Organizing Committee at an early stage. Efficient cooperation with the Olympic Games Organizing Committee partners, the Health Departments inspectors, and the personnel of the coordination center are key factors ensuring effective communication processes and timely response. In future mass gathering events, environmental health programs should aim to successfully deliver environmental health services not only during the Olympic Games, but also during pre-Olympic events.

After the installation of the information exchange network, in January 2004, the inspection grading results were shared with the Athens 2004 Olympic Games Organizing Committee to evaluate the function, reliability and compliance of hotels and Olympic Venues during the test events. Hotels whose inspection results were graded as unsatisfactory or those that did not take the recommended corrective actions for water quality improvement were not used as accommodation sites. Most of the inspected businesses improved their environmental health-related issues significantly by showing compliance with regulations and following the instructions for water quality improvement, probably to avoid negative publicity and aggravating financial consequences[25]. This is a strong indication that the inspection grading system may be an effective tool to motivate change within the industry in order to protect public health[25]. Furthermore, an advantage of repeated standardized grading inspections conducted by different inspectors and registration of inspection results is the reduction of the possibility of corruption. After the successful establishment of the information exchange network, no other disease outbreak was recorded[21,22]. It is possible that single small outbreak incidents may have failed to be reported, but that seems unlikely due to the comprehensive disease surveillance during the test events and the Olympic Games[21,22].

Although insufficient infrastructure and limited time did not affect the effectiveness of the program, the delays of the venues' construction works, and the failure to provide timely funding of the environmental health preparations, deferred the implementation of the program. About 12.7% (5724) of all premises of public health interest located in the five official Olympic cities were inspected within a two-year period of time. Planning for the public health system in Atlanta took more than 2 years,[6] and in Sydney it took almost three years[7]. For future mass gathering events, timely obtainment of appropriate financial resources and construction of venues will allow sufficient time to specify and test the environmental health surveillance system.

Systematic efforts are being made to utilize the heritage left by the 2004 Olympic Games in the field of Environmental Health. Improved liaison between public health agencies and the Ministry of Health and Social Solidarity has yielded ongoing benefits. For the first time a large number of environmental health inspectors from different agencies worked closely together. As in the Sydney Olympic Games, the result was a better understanding of respective roles and responsibilities, and a lasting legacy of efficient network associations[8]. Data concerning registrations, inspections and microbiological test results originating from premises outside the Olympic Venues were available by the PDPH after the Olympic Games, while post event data could be added. Public health preparedness for mass gathering events provides opportunities for national public health programs to enhance infrastructures and improve compliance with public health regulations, provided legal and governmental support is available. The national public health system of the Olympic Games host country can be enhanced by building on procedures used during the Olympic Games preparations.

The experience of the 2004 Olympic Games left the port health authorities well prepared for the challenges posed to ships making their way to Athens, as happened in the Sydney Olympic Games[18]. The large number of cruise ships that converged in Athens for the 2004 Olympic Games presented a unique opportunity to develop and test a comprehensive system for environmental health surveillance on these vessels. The Athens experience is expected to provide valuable information contributing to the future planning of a European Vessel Sanitation Program.

Within western lifestyle settings, the crowded conditions in overpopulated towns, the frequent organization of major sports[10], recreational[26,27], religious, or political events, and the accumulation of large numbers of tourists in vacation destinations of limited capacity especially during the summer, are conditions that can be characterized as mass gatherings[2,3]. Under the continuous pressure of comprehensive, and sometimes exaggerated media coverage, and being aware of the disastrous consequences the failure of the Official Public Health Services might have on the well being of a huge number of people, almost fastidious preparations and precautions were considered imperative before and during the Olympic Games. An integrated environmental health surveillance program as the one implemented during a special event such the Olympic Games is also needed to protect public health on a routine basis, but with a different perspective.

Registration, standardization, computerization of the program, and communication through electronic networks can be included in a continuous national environmental health surveillance program. Registration and conducting of standardized inspection and communication through any electronic medium among public health agencies are essential components of a system functioning on a routine basis. A routine program could include more simple and concise registry forms, while standardized inspection reports could be short and including fewer items. The data collection, analysis and reporting could be on a weekly basis and not daily. Moreover the response system with the standardized corrective action could be peripheral and not central. Premises could be categorized with different priorities for inspection according to the risk. Food premises serving potentially hazardous food could have higher priority for inspection than those serving coffee, carbonated beverages or snacks such as chips, nuts and pop corn. Moreover, premises located to crowded places, or assessed by high-risk population (patients, children etc), or have a history of unsatisfactory inspection results, or not complying with corrective actions, could be inspected more frequently.

As in the case of a functional communicable disease surveillance system, which provides "information for action", environmental health surveillance information should be a crucial instrument for public health decision-making. Environmental health surveillance data could provide information for priority setting, policy decisions, planning, implementation, and evaluation. It can be a tool for prediction and early detection of environmental health hazards in every day operations.

## Conclusion

This article presents information and provides useful conclusions that can be utilized in environmental health management for other mass gatherings as well as routine national health programs. Integration of all issues of environmental health, including the application of emerging technology, is a new aspect that can be applied. The value of timely implementation and installation of communication processes, rapid and coordinated response to unsatisfactory results, and setting priorities adapted to local circumstances are lessons learned for future Olympic Games. The contribution of an efficient Environmental Health Management Program is indispensable for the success of a great event such as the Olympic Games and every effort should be made to provide safe environmental health services during future events. Enhanced environmental health surveillance aimed at public health decision-making, needs to be incorporated in current national public health programs, but with a different perspective than that of mass gatherings.

## Abbreviations

OPU – Olympic Planning Unit

PDPH – Prefecture department of public health

CDC – Centers for Disease Control and Prevention

## Competing interests

The author(s) declare that they have no competing interests.

## Authors' contributions

CH and JK conceived of the study, reviewed the manuscript and made significant comments. VM and JC drafted the manuscript and carried out the data collection, VV, CK, AVo, IK, PP, and AVa carried out the data collection. VD participated in the design of the study and in its coordination. All authors read and approved the final manuscript.

## Pre-publication history

The pre-publication history for this paper can be accessed here:



## Supplementary Material

Additional file 1 The standardized registry form used to register via on-site visits the food premises is attached as additional file. Similar registry forms were used to register other premises such as swimming pools, cooling towers etc. Click here for file

## Figures and Tables

**Table 1:** Inspection results in four periods before and during the Olympics

		**Inspection Results**							
**Standardized inspection forms**	**Inspections**	**January – December 2003**		**January – May 2004**		**June – July 2004**		**Olympic Games**	
		A,* B†	**C‡ (%)**	**A, B**	**C (%)**	**A, B**	**C (%)**	**A, B**	**C (%)**

Food premises	1648	243	45 **(15.6)**	376	93 **(19.8)**	545	53 **(8.9)**	251	42 **(14.3)**
Canteens	20	2	12 **(85.7)**	2	3 **(60.0)**	0	0 (0.0)	0	1 (100.0)
Hotels	883	169	7 **(4.0)**	229	86 **(27.3)**	268	28 **(9.5)**	92	4 (4.2)
Ships	48	0	0 (0.0)	0	12 **(100.0)**	8	2 **(20.0)**	26	0 (0.0)
Swimming pools	374	79	15 **(16.0)**	62	7 **(10.1)**	128	7 **(5.2)**	76	0 **(0.0)**
Water supply systems	1214	189	13 **(6.4)**	354	33 **(8.5)**	314	35 **(10.0)**	275	1 **(0.4)**
Cooling towers	136	18	23 **(56.1)**	7	8 **(53.3)**	37	11 **(22.9)**	32	0 **(0.0)**
Decorative fountains	336	74	156 **(67.8**)	26	14 **(35.0)**	38	7 **(15.6)**	21	0 **(0.0)**
Ice production plants	3	0	3 (100.0)	0	0 (0.0)	0	0 (0.0)	0	0 (0.0)
Areas requiring pest control	171	22	19 **(46.3)**	21	11 **(34.4)**	30	3 **(9.1)**	64	1 **(1.5)**
Waste management facilities	571	146	18 **(11.0)**	89	8 **(8.2)**	91	2 **(2.2)**	215	2 **(0.9)**
Public toilets	3051	185	24 **(11.5)**	603	52 **(7.9)**	661	44 **(6.2)**	1437	45 **(3.0)**
Sewage treatment units	20	7	1 **(12.5)**	3	0 **(0.0)**	6	1 **(14.3)**	2	0 **(0.0)**
Bottled water plants	21	3	3 **(50.0)**	7	0 **(0.0)**	6	2 **(25.0)**	0	0 **(0.0)**
Camps	4	2	0 (0.0)	0	0 (0.0)	1	0 (0.0)	1	0 (0.0)
Seacoast	44	6	0 **(0.0)**	6	10 **(62.5)**	20	0 **(0.0)**	2	0 **(0.0)**
Marinas	18	0	0 (0.0)	1	0 (0.0)	1	0 (0.0)	16	0 (0.0)
**Total**	**8562**	1145	339 **(22.8)**	1786	337 **(15.9)**	2154	195 **(8.3)**	2510	96 **(3.7)**

*Satisfactory inspection result, †Relatively satisfactory inspection result, ‡Unsatisfactory inspection result

**Table 2:** Water Microbiological test results in four periods before and during the Olympics

		**Microbiological test results**							
**Premises**	**Samples**	**January – December 2003**		**January – May 2004**		**June – July 2004**		**Olympic Games**	
		**Total**	**Positive (%)**	**Total**	**Positive (%)**	**Total**	**Positive (%)**	**Total**	**Positive (%)**

Water supply systems*	1657	256	30 **(11.7)**	491	81 **(16.4)**	610	78 **(12.7)**	300	38 **(12.6)**
Water supply systems**†**	2142	292	32 **(10.9)**	767	101 **(13.1)**	870	178 **(20.4)**	213	36 **(16.9)**
Swimming pools	560	123	16 **(13.0)**	66	6 **(9.0)**	170	31 **(18.2)**	201	21 **(10.4)**
Cooling towers	204	81	37 **(45.6)**	15	0 **(0.0)**	64	22 **(34.0)**	44	7 **(15.9)**
Decorative fountains	151	78	0 **(0.0)**	32	2 **(2.5)**	36	0 **(0.0)**	5	0 **(0.0)**
Bottled water plants	252	98	0 **(0.0)**	105	0 **(0.0)**	34	0 **(0.0)**	15	0 **(0.0)**
Seacoast	58	11	2 **(18.1)**	27	0 **(0.0)**	16	0 **(0.0)**	4	0 **(0.0)**
**Total**	**5024**	939	117 **(12.4)**	1503	190 **(12.6)**	1800	309 **(17.1)**	782	102 **(13.0)**

*Samples tested for the presence of coliform bacteria, *Escherichia coli*, intestinal enterococci, *Clostiridium perfringens *(including spores).

**† **Samples tested for *Legionella *spp.
