# Supplementary material for: Management of environmental health issues for the 2004 Athens Olympic Games: is enhanced integrated environmental health surveillance needed in every day routine operation?
Source: BMC Public Health. 2006 Dec 18;6:306. doi: 10.1186/1471-2458-6-306 (PMC1764887; doi:10.1186/1471-2458-6-306)
Supplement: Additional file 1 — The standardized registry form used to register via on-site visits the food premises is attached as additional file. Similar registry forms were used to register other premises such as swimming pools, cooling towers etc. [file 1471-2458-6-306-S1.doc]

REGISTRY FORM FOR FOOD PREMISES

General information about the block

Name : ……………………………………………………………………..… Code: __ __ __ __ __ __ __ __ __ __ __

Prefecture Public Health Department : …………………… Municipality: …………………………………………

Address: …………………………………………………………………………

Tel.: ……………………….… Fax: …………………. E-mail: ……………………………………………………..

Responsible person: ………………………………………………….. Tel: ………………………………..

Olympic Venue: ٱYes ٱNo If Yes, please specify:……………………………………………………….

**Information about the food premise**

Name: ………………………………………………………….. Α.Φ.Μ.: ………………………………………..

Code: __ __ __ __ __ __ __ __ __ __ __ __ __ __ __ __ __ __ __ __

Responsible person for the food premice: ……………………………………………….…………………………………

Tel.: ……………………………………………… Fax: …………………….. E-mail: …………………………….…

Is the food premice included in a shopping precinct? ⬜Yes ⬜No

Is the food premice a chain store? ⬜Yes ⬜No If Yes, please specify: ……………………………………...

Food premice description:………………………………………………………………………..……………………

(e.g. restaurant, canteen)

Permit number: ……………………………….. Authority: ……………………………….……

**Location**: ⬜Basement ⬜Ground floor ⬜Loft ⬜Floor If Yes, please specify: ……

**Capacity:** **Indoors:** ____ **Outdoors:** ____

**HACCP:** ⬜Yes ⬜No

No of food handlers : ___ ___ ___ Floor area: _______ m^2^

**Type of ventilation system**: ……………………………………….

**Fire protection system**: ……………………………………….

**Potable water sourse**: ⬜Municipal water supply ⬜Drill ⬜Spring water ⬜Other, please specify…………

**Drain system**: ⬜Main drainage ⬜Septic tank ⬜Other, please specify…………

No of toilets for customers: M: ___ ___ F: ___ ___ M/F: __ __

No of hand basins: ___ ___ ___

No of toilets for personnel: Α: ___ ___ Θ: ___ ___ M/F: __ __

No of hand basins: ___ ___ ___

No of swimming pools __ __ If any, *Please complete the Registry form for swimming pools*

1. Code of swimming pool: __ __ __ __ __ __ __ __ __ __ __ __ __

2. Code of swimming pool: __ __ __ __ __ __ __ __ __ __ __ __ __

No of decorative fountains __ __ If any, *Please complete the Registry form for* *decorative fountains*

1 Code of decorative fountain: __ __ __ __ __ __ __ __ __ __ __ __ __ __ __

2. Code of decorative fountain: __ __ __ __ __ __ __ __ __ __ __ __ __ __ __

Comments:………………………………………………………………………………………………………………...

Date: …..…/……../…………

Name of person who completed the questionnaire:…………………… Signature:
